# DNA-encapsulated silver nanodots as ratiometric luminescent probes for hypochlorite detection

**DOI:** 10.1186/1556-276X-9-129

**Published:** 2014-03-19

**Authors:** Soonyoung Park, Sungmoon Choi, Junhua Yu

**Affiliations:** 1 Department of Chemistry Education, Seoul National University, 1 Gwanak-ro, Gwanak-gu, Seoul 151-742, South Korea

**Keywords:** Ratiometric luminescent probes, Silver nanodots, DNA, Hypochlorite, Oxidants, Cleaners, Mechanism, Spectral shift

## Abstract

DNA-encapsulated silver nanodots are noteworthy candidates for bio-imaging probes, thanks to their excellent photophysical properties. The spectral shift of silver nanodot emitters from red to blue shows excellent correlations with the concentration of reactive oxygen species, which makes it possible to develop new types of probes for reactive oxygen species (ROS), such as hypochlorous acid (HOCl), given the outstanding stability of the blue in oxidizing environments. HOCl plays a role as a microbicide in immune systems but, on the other hand, is regarded as a disease contributor. Moreover, it is a common ingredient in household cleaners. There are still great demands to detect HOCl fluxes and their physiological pathways. We introduce a new ratiometric luminescence imaging method based on silver nanodots to sensitively detect hypochlorite. The factors that influence the accuracy of the detection are investigated. Its availability has also been demonstrated by detecting the active component in cleaners.

**PACS:**

82; 82.30.Nr; 82.50.-m

## Background

Developing bright luminescent probes is still one of the targets for achieving better optical imaging quality [1,2]. With respect to cellular imaging, the combination of a specific targeting group and the selective response to an analyte is the key to an effective probe design [3,4]. Even though numerous bio-imaging probes have been developed in the last few decades [5], the organic fluorophores used for signaling still suffer from low probe brightness, poor photostability, and oxygen bleaching [6,7]. Consequently, the creation of fluorophores with improved photophysical properties is still in high demand [1,2]. Semiconductor quantum dots (QDs), on the other hand, have been produced to overcome the drawbacks of organic fluorophores [2,8], but they are not sufficiently biocompatible due to their large size, intermittent photon emission, and potential toxicity [9]. Silver nanodots (AgNDs), however, are one of the most notable alternatives to current fluorophores.

AgNDs are small, few-atom clusters that exhibit discrete electronic transitions and strong photoluminescence [10,11]. After the report of the first stable silver nanodots in aqueous solution in 2002 [12], many scaffolds have been developed, for example, based on poly(acrylic acid) [13] or short peptides [14], which stabilize the reduced silver atoms. Among these scaffolds, DNA stabilization has induced the best photophysical characteristics of AgNDs, such as high molar extinction coefficients, high emission quantum yields, and noticeably high photostability. For these reasons, DNA-encapsulated AgNDs have been attracting huge attention in molecular imaging/bio-imaging [10,15-21].

In our previous studies, it has been shown that polycytosine-protected AgNDs (C24 AgND) with red emissions (red emitters, *λ*_em_ = 625 nm) are sensitive to reactive oxygen species (ROS). The oxidization of red emitters by ROS results in yellow (*λ*_em_ = 562 nm) and blue (*λ*_em_ = 485 nm) silver nanodot emitters that show outstanding stability in oxidizing environments. These characteristics make silver nanodots useful as agents for oxidant-resistant imaging and ratiometric luminescence detection [22], which minimizes adverse effects due to the varied probe concentration and other environmental factors that are common in single-wavelength fluorescent detection [23].

Hypochlorite (OCl^−^) is a major ROS species. Especially in immunological cells such as neutrophils, macrophages, and monocytes, cellular OCl^−^ is synthesized by myeloperoxidase (MPO)-catalyzed oxidation of chloride ion with hydroperoxide (H_2_O_2_) [24,25]. The regulated generation of OCl^−^ plays a predominant role during the microbicidal process in the immune system. However, uncontrolled overproduction of OCl^−^ in phagocytes is regarded as a provoking cause of diseases such as Alzheimer's disease [26], atherosclerosis [27], neurodegenerative disease, cardiovascular disease [28], and cancer [29-31]. Even though it is very important and urgent to explain the pathways of OCl^−^ generation and its systemic impact, progress is still slow since it is hard to detect transient ROS refluxes [1,28]. Sodium hypochlorite is also one of the major active ingredients used as a disinfectant and bleach in some cleaners, together with surfactants, builders, solvents, etc. [32]. Even though widely used, excessive hypochlorite may induce neurodegeneration, endothelial apoptosis, ocular irritation, and other tissue damage [24,33-37]. Chemosensors are indispensable to allow us to obtain the exact concentration of OCl^−^ with high spatiotemporal resolution. Organic molecules are still the major fluorescent probes for OCl^−^[38-40], though suffering from their above mentioned drawbacks [28,41]. We were inspired to develop a different class of OCl^−^ probe using our oxidative DNA-encapsulated AgNDs. Prior to evaluating the bio-suitability of our probe, in this report, we investigated the parameters for accurate detection of hypochlorite and evaluated the derived ratiometric imaging method by monitoring the concentration of OCl^−^ in commercially available cleaners.

## Methods

### Chemicals

Silver nitrate (99.9999%), Triton X-100, sodium sulfate, sodium hypochlorite, hydrogen peroxide, starch, sodium thiosulfate, and sodium borohydride were purchased from Sigma-Aldrich (St. Louis, MO, USA) and used as received. DNA was purchased from IDT DNA (Coralville, IA, USA).

### Preparation of silver nanodots

Different silver nanodot emitters were prepared according to published data [15,18,42]. Briefly, single-stranded DNA (ssDNA) and silver ions were mixed at a DNA base/Ag^+^ ratio of 2:1 and reduced with sodium borohydride. Silver nanodots were used as probes 15 h after the chemical reduction of the mixture.

## Results and discussion

**Figure 1:**
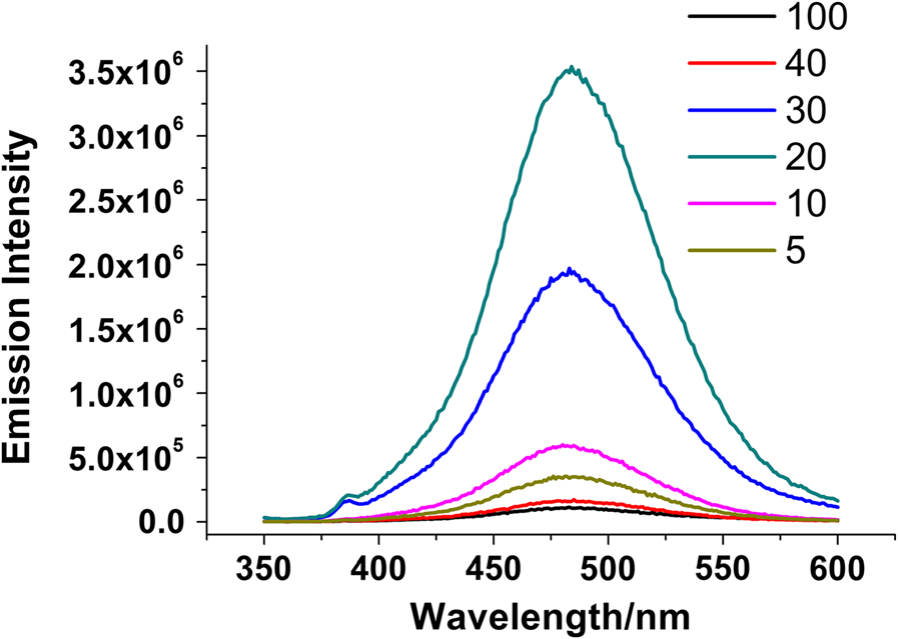
**The influence of sodium borohydride concentration on the formation of blue emitters.** To a C24-Ag solution (50 μM, 1 mL), varied volumes of aqueous sodium borohydride solutions (1 mg/mL) were added. The solutions were left overnight at room temperature to achieve stable red emissions, and then hydrogen peroxide was added with a final concentration of 5 mM. An emission intensity of 340 nm excitation was recorded 5 h later. The numbers indicate the volume of aqueous sodium borohydride solution in microliters.

Upon the reduction of silver ions with borohydride in the presence of single-stranded DNA molecules, a red emission species usually appears. It shifts gradually to the blue emission species, which is considered to be a multistep, intermediate-involved process. Reactive oxygen species expedite the spectral shift by quenching the red emission and facilitating the formation of the blue [22]. The peak shift depends on the concentration of oxidizing agents, which suggests that the remaining borohydride used as a reducing agent for silver nanodot preparation may weaken the oxidizing capacity of oxidants. The amount of borohydride was optimized to produce maximum blue emitters. The mixture of ssDNA and silver ions was reduced with a varied volume of aqueous sodium borohydride solution, followed by the addition of an oxidizing agent. An emission intensity at 340 nm excitation was recorded. The solution with 20 μL of sodium borohydride, corresponding to a Ag^+^/NaBH_4_ ratio of 6:5, yielded the maximum production of blue emitters, slightly lower than the regular NaBH_4_ dose (Figure 1). Too little sodium borohydride led to poor nanodot generation, whereas too much sodium borohydride weakened the oxidizing capacity of hydrogen peroxide.

**Figure 2:**
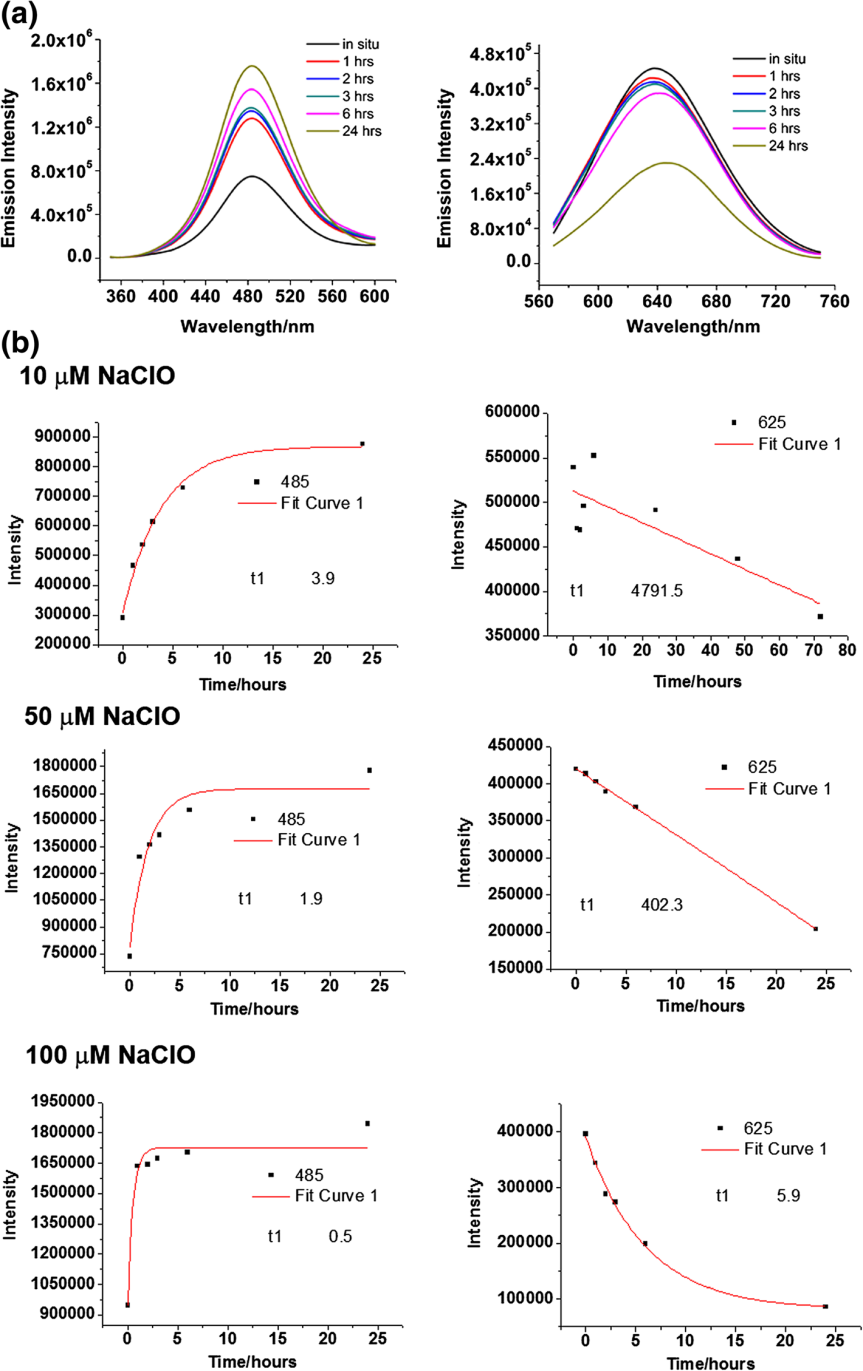
**Reaction kinetics between red silver nanodots and sodium hypochlorite. (a)** Upon the addition of NaClO (50 μM), the red emission was quenched slowly (right), but the blue emission increased fast (left). **(b)** The time course of C24-Ag silver nanodot emissions in the presence of 10, 50, and 100 μM of sodium hypochlorite. 485 and 625 indicate the wavelength at which the intensity was monitored. The red curves are tentative monoexponential fits of the time courses. The fitting indicates that the red emitters degraded much slower than the generation of the blue emitter.

The photoresponses of a 24mer polycytosine-protected silver nanodot (red emitter, *λ*_em_ = 625 nm) upon the addition of sodium hypochlorite (NaOCl) are illustrated in Figure 2, in which the generation of the blue was much faster than the chemical bleaching of the red, with a pseudo-first-order rate constant of 2.5 × 10^−1^ s^−1^ (the blue) versus 2.1 × 10^−4^ s^−1^ (the red). As the concentration of hypochlorite was increased, the difference narrowed between the reaction rates of bleaching and the growth of the nanodots (Figure 2). It is possible that the minor part, but not the major part, of the oxidized species from the red emitter, such as silver ions, contributed to the creation of the blue emitter in this case. The higher the concentration of the hypochlorite, the greater the oxidation of the red emitter.

**Figure 3:**
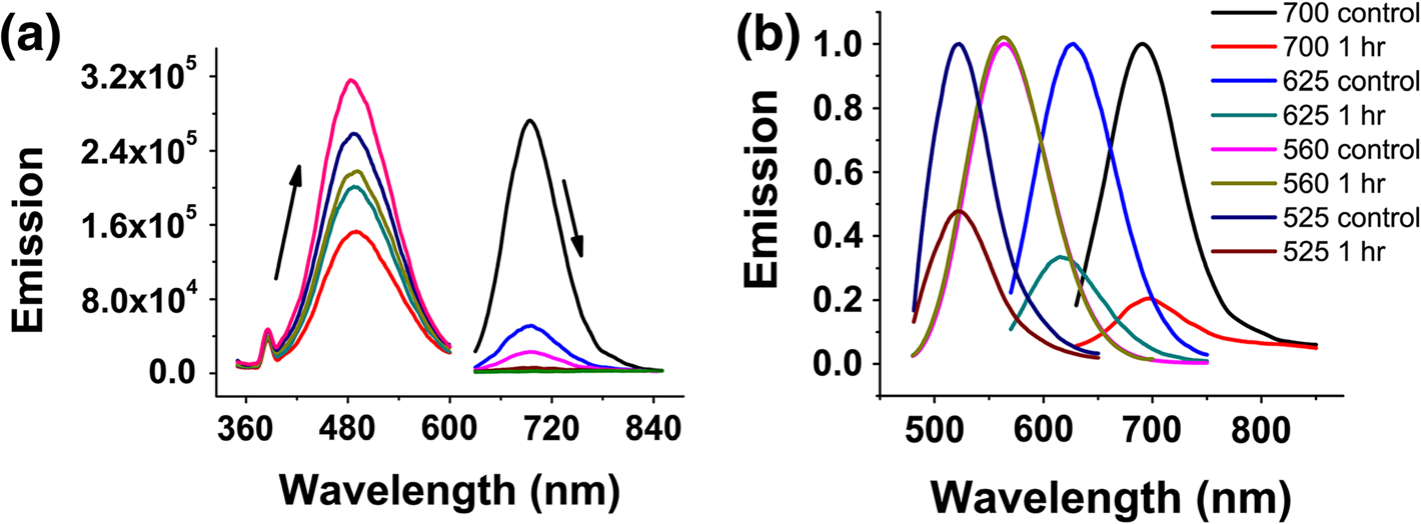
**Comparison of the chemical stability of several silver nanodots towards oxidants. (a)** The spectral shift of the near-IR emitter in the presence of oxidants. **(b)** The emission spectra of the near-IR, the red, yellow, and green emitters were recorded in the absence (marked as control) and presence (marked as 1 h) of oxidants.

Interestingly, several other species showed different stability over oxidants. The near-IR emitter (*λ*_em_ = 700 nm, CCCTAACTCCCC-protected silver nanodot) [15] also exhibited an oxidization pattern (Figure 3a) similar to the red emitter, except for being more sensitive to oxidants. Its emission intensity decreased 80%, compared to a 67% decrease for the red emitter (Figure 3) under the same conditions. However, the yellow emitter (*λ*_em_ = 560 nm, ATATCCCCCCCCCCCCATAT-protected silver nanodot) was much more stable. Its emission intensity decreased less than 1% with a half-life of 35 h, but still shorter than that of the blue (100 h). The green emitter (*λ*_em_ = 523 nm, 20mer polycytosine-protected silver nanodot) [18], however, broke the trend of stability that silver nanodots become more stable when their emission wavelengths shorten, but was still more stable than the red emitter. Contrary to the red and the near-IR emitters, there was no new peak formed in the presence of oxidizing agents for the yellow and green emitters. This might suggest that the blue, green, and yellow species share similar but not identical structural characteristics (e.g., cluster sizes), in which these nanodots present their minimum, inconvertible functional units. After the reduction of silver nitrate in the presence of protection groups, both silver clusters and silver nanoparticles are formed with a wide range of size distributions. When prepared in this way, the absorption spectrum shows not only the typical absorption from spherical silver nanoparticles, but also the absorption of small clusters. Such clusters are small since they cannot be spun down with a high-speed centrifuge. Not all the clusters exhibit photoluminescence (therefore called non-emissive species), while the red and near-IR, together with other non-emissive species stable in a more reducing environment, have to be oxidized or reorganized to intermediates to form nanodots with shorter emission wavelengths. The oxidation of precursors of yellow and green emitters (both are red emitters) in stronger oxidizing environments resulted in only blue emitters, which suggests that the formation of the yellow and the green requires more sophisticated rearrangements than the blue. Strong oxidizing environments transfer the red precursors unidirectionally to intermediates only suitable for the blue formation, likely in smaller sizes due to faster oxidation.

Blue emission intensity leveled off kinetically at a certain point and decreased gradually (Figure 2). The turning point depended on the concentration of hypochlorite. Generally, higher concentrations of oxidants did not increase the maximum blue emission intensity but just accelerated the transfer to the blue, leading to a fast response time towards the detection of oxidants. A trade-off between blue emitter stability and detection sensitivity suggested that the effective detection range was 1 to 120 μM for sodium hypochlorite [22].

**Figure 4:**
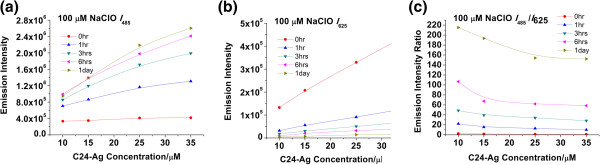
**Emission and emission ratios of C24-Ag silver nanodots in the presence of 100 μM of sodium hypochlorite.** Emission was examined after the addition of an oxidant to the nanodot solutions. The higher the concentration, the stronger the emissions at **(a)** 485 nm and **(b)** 625 nm. However, **(c)** the *I*_485_/*I*_625_ ratios at varied concentrations showed much less fluctuation at a given concentration of the oxidizing agent.

One of the advantages of ratiometric detection is its tolerance to the variation in probe concentration. Usually, the emission intensity is proportional to the silver nanodot concentration. The higher the concentration, the stronger the emissions at 485 and 625 nm (Figure 4a,b). However, the *I*_485_/*I*_625_ ratios showed much less fluctuation at a given concentration of the oxidizing agent when the nanodot concentration varied between 15 and 35 μM (Figure 4c), indicating that the silver nanodot concentration had little impact on the detection accuracy of the hypochlorite concentration.

**Figure 5:**
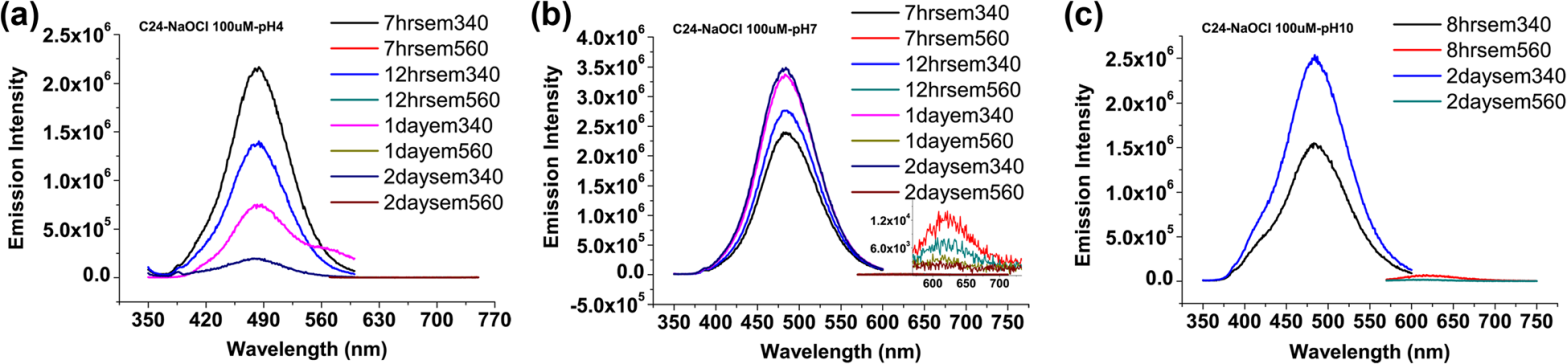
**Influence of pH on oxidization and stability of C24-Ag silver nanodots in presence of 100 μM sodium hypochlorite.** The emission intensity of 485 nm decreased at pH = 4 **(a)** but gradually increased at pH = 7 **(b)** and pH = 10 **(c)**. The numbers before ‘hrs’ or ‘day’ in the legends indicate the time at which the emission was measured, and those after the ‘em’ indicate the excitation wavelengths.

Since the intensity ratio of the blue/red strongly depends on reaction kinetics between silver nanodots and oxidants, some factors, such as pH and temperature, will influence the reaction rates. As we mentioned earlier, whether it is suitable as a probe in physiological pH is an important factor in successfully measuring OCl^−^ in bio-organisms. Our results (Figure 5) suggested that neutral solutions assisted consistent results. In this study, all the detections of oxidants were conducted in pH 7 solutions at 25°C, which are potentially useful for further *in vivo* probe designing.

Sodium hypochlorite is used widely in some cleaners as a disinfectant and bleach. To accurately detect the hypochlorite concentration in household cleaners *in vitro*, we examined the influence of some salts and surfactants on the photoresponse of silver nanodots. Since many cleaners contain sodium salts and sulfonate [43], we chose sodium sulfate as a basic builder in the calibration buffer for hypochlorite detection. The intensity of emissions of nanodots was lower as the sodium sulfate concentration increased from 100 to 10 mM, but the ratios of blue/red emission intensity were similar. Some surfactants, such as saturate aqueous polyvinyl alcohol solution, did not change the photophysical properties of silver nanodots. Triton X-100, on the other hand, facilitated the generation of the blue emitter slightly but had little influence on the red emitter until the concentration reached 50 mM.

**Figure 6:**
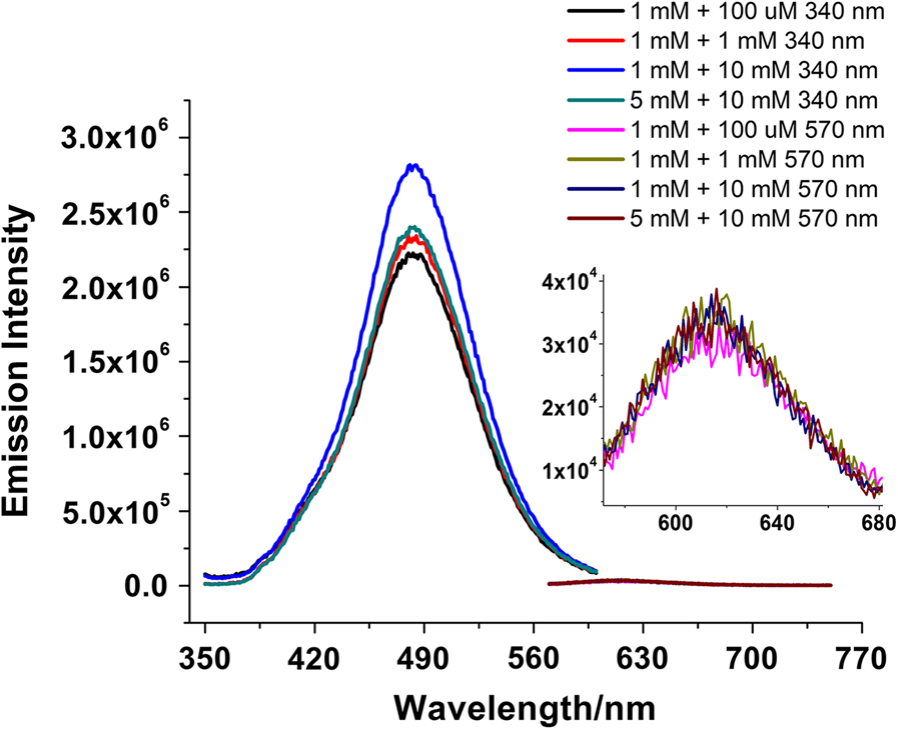
**Combinations of varied concentrations of sodium sulfate and Triton X-100 in a sodium hypochlorite solution (100 μM).** The left peaks were excited at 340 nm and the right at 560 nm. The inset is a close-up of the red peaks. The left numbers in the legend indicate the concentration of sodium sulfate and the right the concentration of Triton X-100.

However, several combinations of sodium sulfate and Triton X-100 at various concentrations showed a *I*_485_/*I*_625_ ratio of 85 with a standard error of 3 after a 5-h incubation in the presence of sodium hypochlorite (100 μM), indicating that the components of the above mixture would not interfere much with the photoresponses of silver nanodots towards hypochlorite (Figure 6).

**Figure 7:**
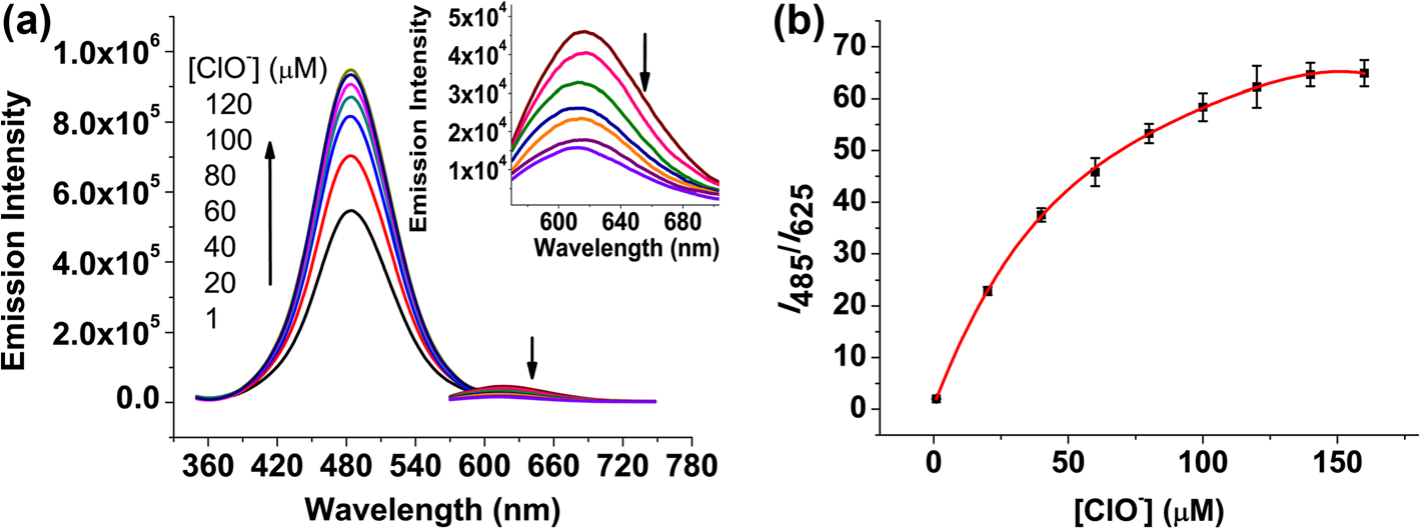
**Luminescence titration of red silver nanodots with sodium hypochlorite. (a)** Emission spectra were acquired 6 h after hypochlorite addition in 10 mM Triton X-100 and 5 mM sodium sulfate solution at pH 8.3. Inset: A close-up of the red region. **(b)** The plot of luminescence intensity ratio of *I*_485_/*I*_625_ against OCl^−^ concentration. The data was fitted with a fourth-order polynomial function. The error bars represent the standard errors.

We chose four commercially available cleaners of both global and local brands marked A through D. The samples were diluted 6,000-fold into silver nanodot solutions (25 μM, 1 mL). The photoresponses of the nanodots were recorded, and the ratios of emission intensity *I*_485_/*I*_625_ were compared to a calibration curve of C24-Ag nanodots obtained from solutions with 5 mM NaSO_4_ and 10 mM Triton X-100 at varied hypochlorite concentrations (Figure 7).

It should be noted that the plot of luminescence intensity ratio of *I*_485_/*I*_625_ against OCl^−^ concentration was not linear. Instead, it leveled off at a higher hypochlorite concentration, which can be partly explained by the concurrent generation and bleaching of the blue emitter both due to hypochlorite. The higher concentration of hypochlorite especially bleached the blue emitter faster, offsetting the increase of blue emission. Consequently, the detection region below 40 μM of hypochlorite was preferred in terms of better detection sensitivity. These cleaners contained 0.20 to 0.73 M of hypochlorite. Some were lower than the recommended sodium hypochlorite concentrations in household bleach (5.25% to 6.15%) [44]. Our results were verified by redox titrations of these samples based on the OCl^−^/I_3_^−^/starch/S_2_O_3_^2−^ method [45] (Table 1), suggesting that our method is an excellent alternative for easy, fast, and accurate hypochlorite detection.

**Table 1:** Detected hypochlorite concentrations in several commercially available cleaners

**Sample**	**A**	**B**	**C**	**D**
Nanodot method (M)	0.23 ± 0.01	0.73 ± 0.05	0.20 ± 0.02	0.20 ± 0.01
Titration method (M)	0.21 ± 0.01	0.74 ± 0.01	0.20 ± 0.01	0.20 ± 0.01

## Conclusions

In summary, we demonstrated dual-wavelength response silver nanodot emitters with outstanding photophysical properties. The excellent stability of the blue silver nanodots in an oxidizing environment leads to their being formulated as probes to detect hypochlorite ions. In particular, we have investigated the factors that influence the photoresponse of the silver nanodots and demonstrate the availability of nanodots by monitoring the concentration of OCl^−^ inside several commercial cleaners.

## Competing interests

The authors declare that they have no competing interests.

## Authors’ contributions

SC and JY conceived the study and participated in its design and coordination. SP and SC carried out the experiments. SP, SC, and JY drafted the manuscript. All authors read and approved the final manuscript.
